# Inadvertent intravenous administration of maternal breast milk in a six-week-old infant: a case report and review of the literature

**DOI:** 10.1186/1756-0500-7-17

**Published:** 2014-01-08

**Authors:** Michaela Döring, Birgit Brenner, Rupert Handgretinger, Michael Hofbeck, Gunter Kerst

**Affiliations:** 1 Department of Pediatric Hematology and Oncology, University Children’s Hospital Tübingen, Hoppe-Seyler-Str.1, 72076 Tübingen, Germany; 2 Department of Pediatric Cardiology and Pediatric Intensive Care, University Children’s Hospital Tübingen, Hoppe-Seyler-Str.1, 72076 Tübingen, Germany; 3 Department of Pediatric Cardiology, University Children’s Hospital Giessen, Feulegenstr12, 35390 Giessen, Germany

**Keywords:** Woman milk, Intravenous administering, Misconnection, Enteral feeding systems

## Abstract

**Background:**

Accidental intravenous administration of an enteral feeding can be fatal or cause complications such as sepsis, acute respiratory and circulatory failure, acute renal failure, hepatic insufficiency, coagulation disorders and severe permanent neurological sequelae. These “wrong route” errors are possible due to compatible connections between enteral feeding systems and intravascular infusion catheters.

**Case presentation:**

We report a six-week-old male infant who received a 5 ml intravenous infusion of breast milk. Within five minutes of administration the child developed tachycardia and tachypnea, accompanied by a sudden decrease in oxygen saturation on pulse oximetry to 69%. The infant received supplemental oxygen via nasal cannula and was transferred to the pediatric intensive care unit. Broad-spectrum antibiotics were administered for 48 hours. Vital signs returned to normal within a few hours. Neurological follow-up through 3 years did not reveal any neurodevelopmental abnormalities.

**Conclusion:**

Development of specific enteral feeding connections, which are incompatible with intravascular catheter connections, is needed urgently to prevent a misconnection with potential morbidity or mortality of children.

## Background

Accidental intravenous administration of enteral feeding can cause severe complications including sepsis, multi-organ failure and death [1-6]. Tubing and catheter misconnections are an important and underreported problem in healthcare [7]. In addition to peripheral and central intravenous catheters and various feeding tubes, peritoneal dialysis catheters, epidural catheters and tracheostomy cuff inflation tubes also have had misconnections [7-10]. In many cases the outcome was fatal [3,5,7,11]. We report a six-week-old male infant who received 5 ml of breast milk intravenously and recovered completely. We discuss the medical management of this patient, and administrative measures and practices we propose to prevent future incidents.

## Case presentation

Postnatal assessment of a male newborn, delivered at 40 weeks gestation (birth weight 4.065 kg, height 52 cm), revealed malformation of the ribs and vertebral column with duplications of thoracic vertebrae, hypoplasia of the right lung, and partial liver herniation through a small diaphragmatic defect into the right hemithorax. On the 48^th^ day postpartum, a simply herniotomy was performed for a right inguinal hernia. On the first postoperative day feeding was started via a nasogastric tube. To promote mother-infant interaction, the mother was instructed in enteral feeding procedures via the nasogastric tube. However, the mother administered 5 ml expressed breast milk intravenously with a syringe. The infant experienced an immediate decrease of oxygen saturation with pulse oximetry of 69%, developed sinus tachycardia of 195/min and tachypnoea with a respiratory rate of 84/min. Blood pressure remained normal, and oxygen saturation normalized following supplementation with nasal cannula oxygen using flow rates of 3–4 L/min. Due to the imminent risk of microembolism and nonspecific activation of coagulation, the infant was transferred to the intensive care unit. Chest X-ray did not reveal infiltrates, pleural effusions or a pneumothorax. Both an electrocardiogram and echocardiography did not show acute right ventricular overload. Ampicillin was given intravenously for a total of 48 hours. Laboratory values one, six and 12 hours after the incident neither revealed evidence for an infection, renal or hepatic dysfunction, a metabolic or coagulation disorder nor myocardial damage. Cerebral ultrasound was normal two hours after the event. Microbial cultures of expressed breast milk were negative. There was no microbiotic evidence of cytomegalovirus in expressed breast milk. The respiratory rate normalized within 1.5 hours, and the heart rate within 2 hours. Four hours after the incident, supplemental oxygen was discontinued. The infant’s blood pressure and temperature were stable throughout the incident. The child was transferred to the pediatric surgical ward 24 hours after the incident and discharged on the third postoperative day without the nasogastric tube. Follow-up examinations through 3 years of age showed normal neurological development.

### Discussion

There are few reports of medical injury due to catheter misconnections in scientific journals; however, they are only the tip of the iceberg worldwide [5,7]. Through 2006, more than 300 cases of misconnection errors have been reported to the United States Pharmacopeial Convention [5]. These events are often fatal and occur in a wide variety of settings [5,7]. Affecting adults [1-3,6,12-17], infants and children [4,5,18-20]. An important lesson from these reports is that experience does not prevent wrong route errors.

Intravenous infusion of enteral feeding may lead to respiratory [15,18-20], renal [3] or hepatic insufficiency [3,18], diffuse myocardial damage [12], metabolic acidosis [18], coagulation disorder [12], increased production of stool [19,21], anaphylactic reactions [12,21], sepsis [1,2,4,6,13,14,18] and death [3,11,22]. Multi-organ failure may not be linked directly to high output septic shock [3,18]. Seizures and permanent neurological impairment have been described in a preterm infant [18]. Some of the clinical features may be explained by microembolism of fat globules and water insoluble particles, as well as an immune response to foreign antigens [3,12]. Cytomegalovirus infection in infancy caused by intravenously infused contaminated breast milk, bypassing the gastrointestinal barrier, has not been reported, however it is prudent to consider this risk. The composition of the enteral feeding, the volume, and the rate of administration are likely to be related to the severity of symptoms. The rarity of reported cases, however, prevents definite conclusions.

**Figure 1:**
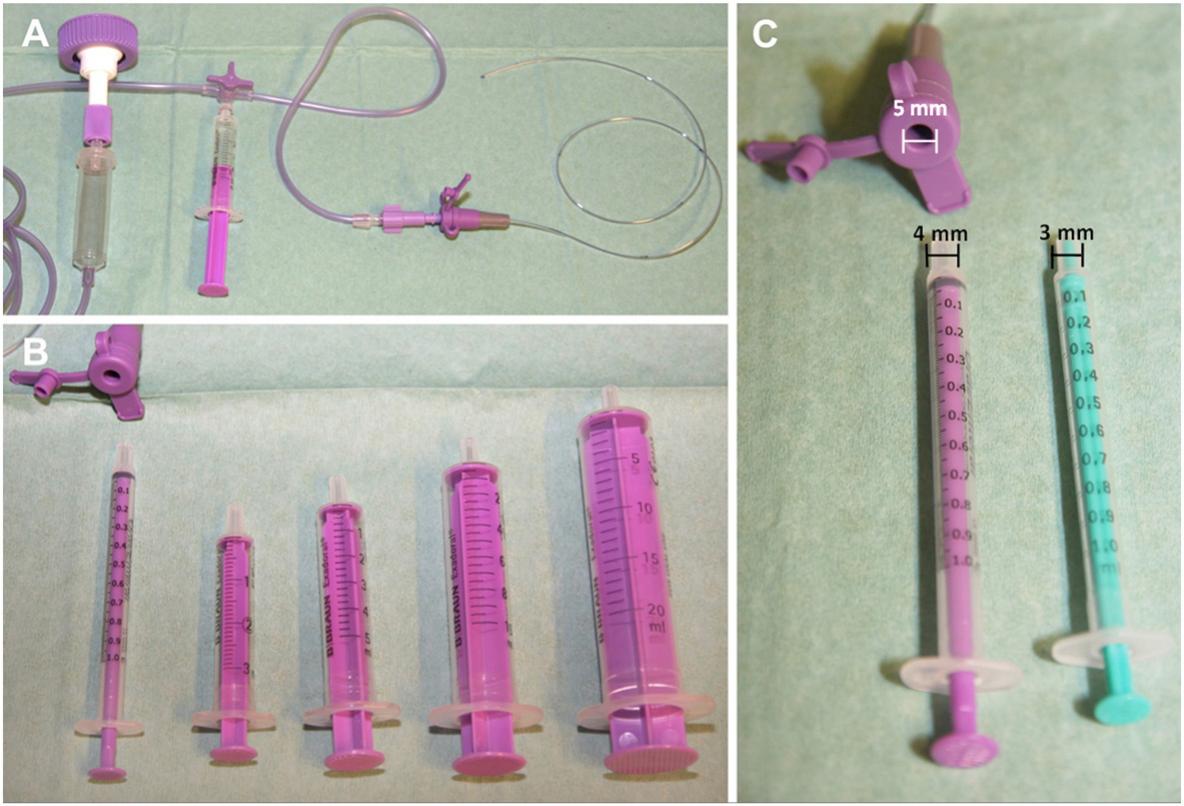
**Specific tubing and equipment for enteral nutrition. (A)** Gastric tubing with specific adapter for connecting enteral feeding tubings with built-in three-way stopcock, to which only specific food syringes can be connected. **(B)** and **(C)** Specific entry of a gastric tubing, which has a larger opening of 5 mm in diameter for connection of specific food syringes of different sizes (1 ml, 3 ml, 5 ml, 10 ml and 20 ml). Food syringes (color purple) have a larger cone with a diameter of 4 mm in comparison to an intravenous syringe (color green) cone (diameter 3 mm) for intravenous infusion systems. The intravenous syringe cone (green syringe) is too small and does not fit into the entry of gastric tubing (color purple). An additional safety feature is the consistent color coding (purple) for all parts used for the application of food.

Management of most cases has been supportive and included oxygen supplementation and mechanical ventilation [3,5,13,18], diuretic therapy [12,23], peritoneal dialysis [3], steroid administration [12,15,19], and circulatory support with fluid resuscitation and catecholamines [1,3,12,13,15,18]. Broad-spectrum antibiotics were administered and then tailored to the results of blood and feeding cultures [1,3,12,13,15,23]. Heparin has been administered in some cases as prophylaxis to prevent thromboembolic events [12,15]. Plasmapheresis and exchange transfusion in an adult and a preterm infant have been reported to improve oxygenation and stabilize hemodynamics, respectively, in an attempt to remove foreign antigens and toxins [13,18]. Since the first report of obvious intravenous administration of an enteral feeding [12], numerous strategies have been proposed to further reduce wrong route errors [4,5,7,15,18,20]. In order to prevent such misconnections, tubings should always be traced to the point of origin connection before any infusion is initiated, including enteral feeds. Enteral feeding syringes and pumps should always be labeled. Education and instruction of all caregivers is necessary. One of the safest preventive measures which has been taken in our hospital after a causality analysis of the reported case, is the use of tubings that are specifically designed for enteral feeding (Figure 1). Government sponsored manufactures standards should identify this situation as solvable at the manufacturing stage and mandate individual non-interchangeable design for intravenous tubing and enteral feeding catheters.

Systematic or institutional practices and procedures should be designed to reduce medical errors, for example, the use of an electronic health record with instructions to avoid errors and the use of Six Sigma as a management system for process improvement [24]. A Hospital Safety Committee consisting of a multidisciplinary group should be established and given the mandate to ensure continuous quality improvement in patient safety [25]. More published reports of such medical errors are required to create guidelines for the optimal course of action to take in such cases.

## Conclusion

Wrong route errors are associated with a high morbidity and mortality risk. Specifically designed non interconnecting tubing should be used to prevent consequences that can arise from misconnected enteral feeding tubing. Medical staff and associated caregivers should be instructed in the use of enteral feeding tubing to prevent route errors.

## Consent

Written informed consent was obtained from the patient’s mother for publication of this Case Report and any accompanying images. A copy of the written consent is available for review by the Editor-in-Chief of this journal.

## Competing interests

The authors declare that they have no competing interests.

## Authors’ contributions

All authors have participated in case report design, interpretation, and writing of the report. MD collected the data of the case report, review of literature, and drafted the first version of the manuscript. BB collected the data of the case report. RH and MH reviewed the manuscript. GK primarily participated in case report design and helped to draft the manuscript. All authors read and approved the final manuscript.
